# A comprehensive mathematical model for cardiac perfusion

**DOI:** 10.1038/s41598-023-41312-0

**Published:** 2023-08-30

**Authors:** Alberto Zingaro, Christian Vergara, Luca Dede’, Francesco Regazzoni, Alfio Quarteroni

**Affiliations:** 1 MOX, Laboratory of Modeling and Scientific Computing, Dipartimento di Matematica, Politecnico di Milano, Piazza Leonardo da Vinci 32, 20133 Milano, Italy; 2 ELEM Biotech S.L., Pier01, Palau de Mar, Plaça Pau Vila, 1, 08003 Barcelona, Spain; 3 LaBS, Dipartimento di Chimica, Materiali e Ingegneria Chimica “Giulio Natta”, Politecnico di Milano, Piazza Leonardo da Vinci 32, 20133 Milano, Italy; 4 Institute of Mathematics, École Polytechnique Fédérale de Lausanne, Station 8, Av. Piccard, CH-1015 Lausanne, Switzerland

**Keywords:** Computational models, Computational biophysics

## Abstract

The aim of this paper is to introduce a new mathematical model that simulates myocardial blood perfusion that accounts for multiscale and multiphysics features. Our model incorporates cardiac electrophysiology, active and passive mechanics, hemodynamics, valve modeling, and a multicompartment Darcy model of perfusion. We consider a fully coupled electromechanical model of the left heart that provides input for a fully coupled Navier–Stokes–Darcy model for myocardial perfusion. The fluid dynamics problem is modeled in a left heart geometry that includes large epicardial coronaries, while the multicompartment Darcy model is set in a biventricular myocardium. Using a realistic and detailed cardiac geometry, our simulations demonstrate the biophysical fidelity of our model in describing cardiac perfusion. Specifically, we successfully validate the model reliability by comparing in-silico coronary flow rates and average myocardial blood flow with clinically established values ranges reported in relevant literature. Additionally, we investigate the impact of a regurgitant aortic valve on myocardial perfusion, and our results indicate a reduction in myocardial perfusion due to blood flow taken away by the left ventricle during diastole. To the best of our knowledge, our work represents the first instance where electromechanics, hemodynamics, and perfusion are integrated into a single computational framework.

## Introduction

Myocardial perfusion is the process by which oxygenated blood is delivered through the coronary arteries to the heart muscle or myocardium, enabling its oxygenation and metabolism. The microvasculature of the myocardium is responsible for facilitating the exchange of oxygen and nutrients with the blood. However, when the coronary circulation is obstructed due to factors such as arterial stenosis or cardiac pathologies like aortic regurgitation and arrhythmias, the blood supply to the cardiac muscle may be limited. This restricted blood flow can lead to ischemia and potentially trigger a myocardial infarction, commonly known as a heart attack^1^.

**Figure 1:**
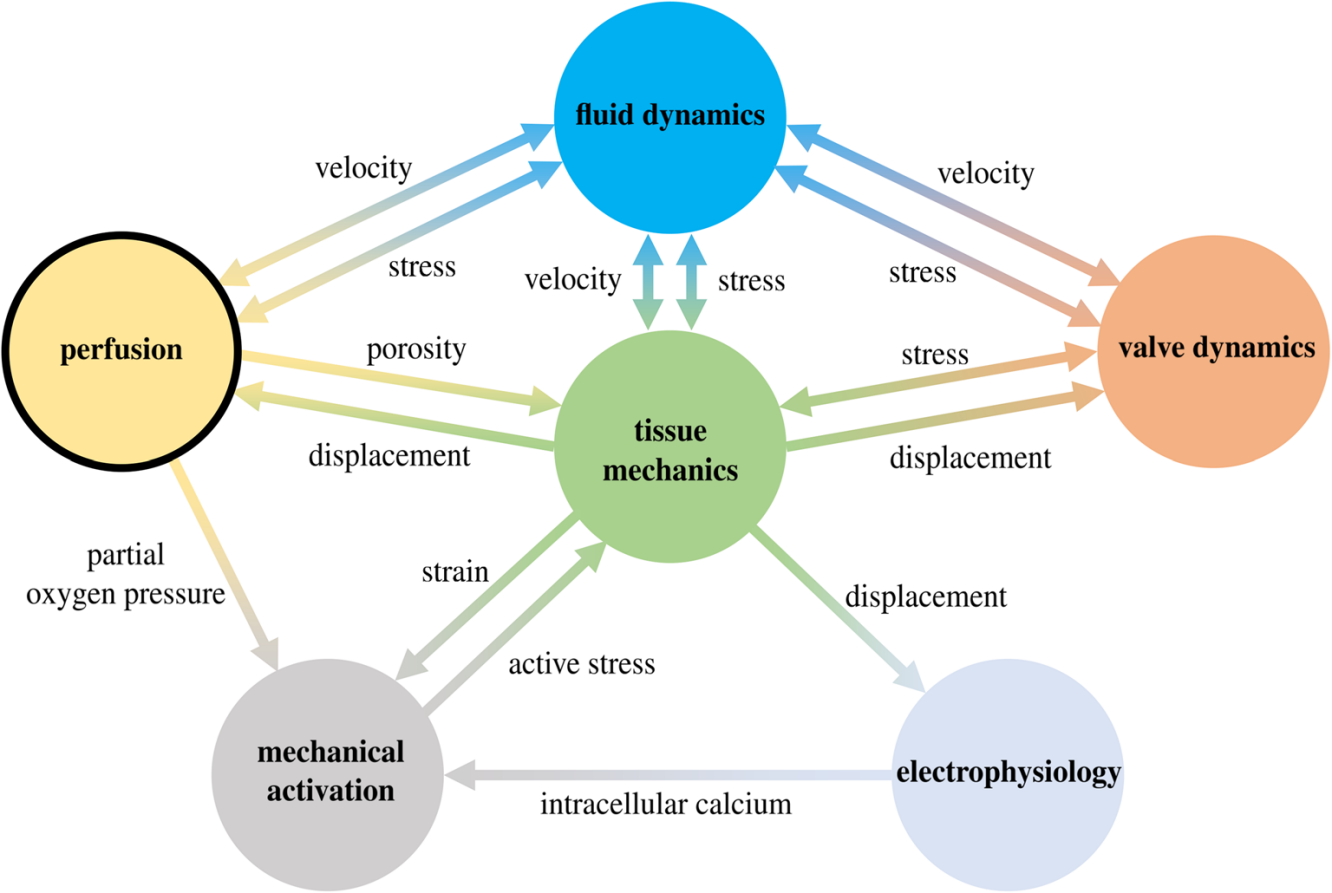
The perfusion is the result of complex interactions among different models.

Stress myocardial computed tomography perfusion (stress-CTP) is a method for quantitatively assessing myocardial blood perfusion through myocardial blood flow maps (MBF), obtained by exposing patients to additional radiation (with respect to standard angiography) and administering an intravenous stressor during a CT scan. *In-silico computational models*^2–4^ can provide valuable insights into physiological processes and enable the simulation of virtual scenarios under multiple pathological conditions, making them useful for studying e.g. coronary by-passes^5^ and ventricular hypertrophy^6^. However, the development of a comprehensive model of myocardial perfusion requires accounting for the complex interactions among multiple physical processes, including the coexistence of multiple spatial scales in the coronary circulation. The coronary arterial tree can be subdivided into *epicardial coronary arteries* (large coronaries) and *intramural vessels* (arterioles, venules and microvasculature)^7^. From a modeling point of view, the blood flow in the large epicardial vessels can be described using full 3D fluid dynamics or fluid-structure-interaction equations^7–12^, or geometrically reduced hemodynamics model, as 1D models^13–15^. Differently, below a threshold length scale, the blood flow in the myocardium can be represented as in a porous medium^16^, thanks to Darcy or multicompartment Darcy models^4,13,17–20^. The integration of these models yields a coupled mathematical problem, featuring dynamic and kinematic conditions at the interface between large coronaries and microvasculature^17,18^.

Figure 1 displays the intricate processes involved in myocardial perfusion, which result from the interplay of various physical phenomena and scales, including electrophysiology, mechanical activation, tissue mechanics, cardiac hemodynamics, and valve dynamics. In this paper, we propose for the first time a novel mathematical model that unifies these different aspects within a single framework. Our mathematical model includes *core models* for electrophysiology, active and passive mechanics, blood fluid dynamics in the left atrium, ventricle, and aorta, mitral and aortic valve dynamics, and myocardial blood perfusion. To partially decouple the problem, we use a fully-coupled electromechanical model to trigger a fully coupled Navier–Stokes-multi-compartment Darcy perfusion model. To the best of our knowledge, this work represents the first attempt in the literature to integrate electromechanics, fluid mechanics, and perfusion into a single computational framework.

To evaluate the biophysical fidelity of the proposed model, we compare our coronary flow rates and average myocardial blood flows with the corresponding clinical ranges documented in the medical literature. The results indicate that our computational model successfully replicates a healthy simulation scenario. In addition, we successfully simulate a severe aortic valve regurgitation, which can cause reduced oxygen delivery to the myocardium due to steal of coronary flow during diastole.

Our novel integrated model is mathematically sound and physiologically accurate, as it does not require any assumptions about boundary conditions at the inlet sections of large coronary arteries, as commonly done for instance in refs.^18,21–24^ . As a matter of fact, in our model, the coronary orifices are internal sections of the 3D computational fluid domain. This also allows us to relax the one-way coupling hypothesis between large vessels circulation and coronaries as done in the context of poromechanics in ref.^20^. Furthermore, differently from previous works on cardiac perfusion modeling, our computational framework features a detailed 3D electro-mechano-fluid model to provide precise inputs for myocardial perfusion. Our computational model enables potential simulations of the effects that various pathologies have on cardiac perfusion, such as the disturbed blood flow due to a valvular disease, a reduced support of flow due to a coronary stenosis, and the effects of an electrical dysfunction down to the microvasculature, to mention a few. To illustrate this aspect, we demonstrate the capability of our model by simulating an aortic regurgitant valve. The accurate results that we obtained have been thanks to the simulation of the entire left heart and the modeling of the aortic valve. These features of our novel proposed model make it substantially different and innovative with respect to the framework introduced in ref.^24^. Our work is a significant advancement towards the realization of an integrated model of the whole human heart function, which would enable in-depth investigations of physiological and pathological perfusion scenarios, including the myocardial ischemia resulting from a coronary artery occlusion.

## Methods

To describe the methodology that we develop for the multiphysics simulation of cardiac perfusion, we first introduce our mathematical model, then we give details on numerical methods, software libraries, and computational setup.

### Mathematical model

For the mathematical model, we consider the time domain (0, *T*) and three different spatial domains:

- The *left heart solid domain* $\Omega ^{\textrm{s}}$, comprising the ventricle and atrial myocardium, and the aortic vessel wall. In $\Omega ^{\textrm{s}}$, we define the electromechanical problem. We consider a Lagrangian framework set in the reference unloaded configuration $\widehat{\Omega }^{\textrm{s}}$.
- The *left heart fluid domain* $\Omega ^{\textrm{f}}$, comprising the left ventricle and atrium chambers, together with the aorta and the epicardial coronaries. In $\Omega ^{\textrm{f}}$, we define the fluid dynamics problem. $\Omega ^{\textrm{f}}$ is a time dependent domain, but we omit the subscript *t* to keep the notation simpler.
- The *biventricular geometry* $\Omega ^{\textrm{p}}$, that we model as if it were a porous medium, where we set our perfusion model. We neglect the effect of tissue deformation on blood perfusion. In practice, this assumption translates into considering the domain $\Omega ^{\textrm{p}}$ non deformable^17–19^.

**Figure 2:**
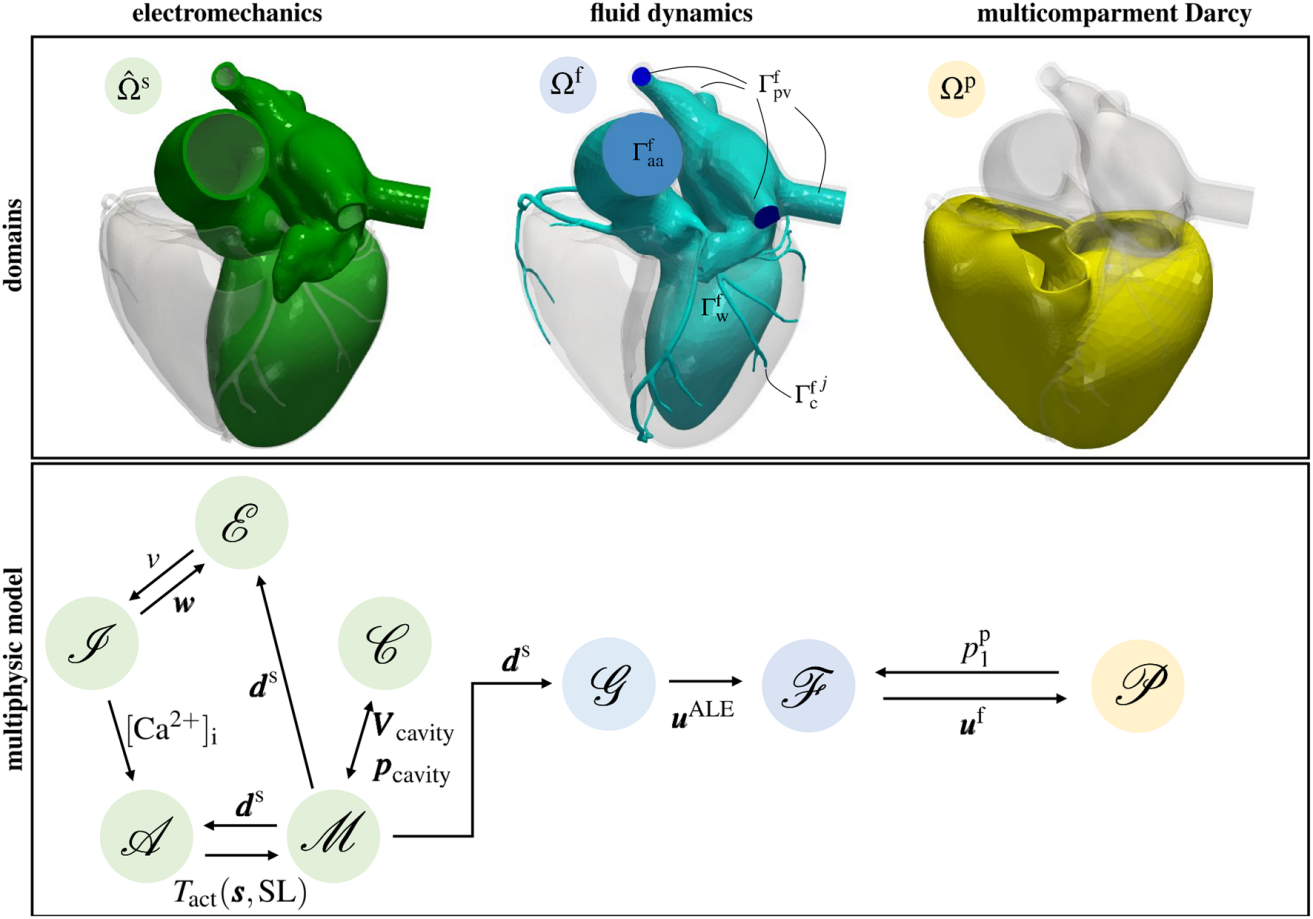
Description of the general layout of the computational model. On the top, computational domains: the left heart solid domain $\widehat{\Omega }^{\textrm{s}}$ in green, the left heart fluid domain $\Omega ^{\textrm{f}}$ in blue, and the biventricular geometry $\Omega ^{\textrm{p}}$ in yellow. On the bottom, coupling of different models: the 3D-0D electromechanics model (in green, where ${\mathscr {E}}$ is the electrophysiology model, ${\mathscr {I}}$ is the ionic model, ${\mathscr {A}}$ is the activation model, ${\mathscr {M}}$ is the mechanical model, and ${\mathscr {C}}$ is the 0D circulation model) is one-way coupled to the fluid dynamics model in moving domain (in blue, where ${\mathscr {G}}$ is the fluid geometry problem and ${\mathscr {F}}$ is the fluid dynamics model) that is fully-coupled to the perfusion model (${\mathscr {P}}$). The different variables are introduced in the “Methods” section.

We give a graphical representation of each domain in Fig. 2, top. Notice that we ignore fluid dynamics in the right heart since coronaries originate from the left heart. Hence, there is not a direct feedback of the right heart hemodynamics on myocardial perfusion. Accordingly, also the electromechanical simulation has been performed in the left heart solely.

In the following, we describe each physical problem occurring in the different domains, and provide details on the coupling conditions. The overall multiphysics model is sketched in Fig. 2, bottom.

#### Electromechanics

To model the electric and mechanical activity of the heart, several mathematical and numerical models have been proposed in the literature^25–32^. We consider the model presented in^33,34^ which is set in the left heart solid domain $\widehat{\Omega }^{\textrm{s}}$ shown in Fig. 2, top. For the recovery of the reference configuration^26,35–37^, we refer specifically to the algorithm presented in^33^. We reconstruct cardiac fibers with the *Laplace-Dirichlet Rule-Based Methods*^38–40^, using the algorithms for ventricles and atria presented in^40^ and^41^, respectively.

We assume that the active mechanics triggered by electrophysiology is present only in the left ventricle $\widehat{\Omega }^{\textrm{s}}_{\textrm{LV}}$. Conversely, since our focus is on the dynamics downstream the aortic valve, we treat the atrial tissue as an electrically passive material. This is a simplification that has been commonly adopted in other electromechanics^31,42–44^ and electro-mechano-fluid^45–47^ models of the heart. This simplification, however, does not hamper the impact on blood flow supplied to the heart muscle. As a matter of fact, the active mechanics of the atria would mainly affect the flow during the relaxation phase of the ventricles, specifically when there is a secondary jet passing through the mitral valve during the A-wave^48^. However, the ventricular flow during the filling phase does not have a major effect on cardiac perfusion.

We model electrophysiology by the evolution of the transmembrane potential *v* in the left ventricle via the *monodomain equation*^49^. We denote the electrophysiology problem in compact form as

1 $$\begin{aligned} {\mathscr {E}} (v; \varvec{w}, \varvec{z}, \varvec{d}^{\textrm{s}}) = 0 \; \qquad \text { in } \widehat{\Omega }^{\textrm{s}}_{\textrm{LV}} \times (0, T), \end{aligned}$$

where $\varvec{w}$ and $\varvec{z}$ are the gating variables and ionic concentrations, respectively. Note that the monodomain equation is augmented with *mechano-electric feedbacks*^50–52^, as highlighted by the dependence from the solid displacement $\varvec{d}^{\textrm{s}}$. We couple Eq. (1) with the *ten Tusscher and Panfilov ionic model*^53^, that we denote in short as

2 $$\begin{aligned} {\mathscr {I}} ( \varvec{w}, \varvec{z}; v) = 0 \; \qquad \text { in } \widehat{\Omega }^{\textrm{s}}_{\textrm{LV}} \times (0, T). \end{aligned}$$

We model the active contractile force^54^ by means of the biophysically detailed *RDQ20 activation model*^55^, which accounts for the force-sarcomere length relationship and the force-velocity relationship thanks to fiber strain-rate feedback, which we deem essential to faithfully predict blood fluxes and velocities in the CFD simulation^34,56^. Denoting by $\varvec{s}$ the state variables related to the active contractile force and by $\textrm{SL}$ the sarcomere length, which depends on the displacement $\varvec{d}^{\textrm{s}}$, we express the activation model in compact notation as

3 $$\begin{aligned} {\mathscr {A}} ( \varvec{s}; [\textrm{Ca}^{2+}]_{\textrm{i}}, \textrm{SL}(\varvec{d}^{\textrm{s}})) = 0 \; \qquad \text { in } \widehat{\Omega }^{\textrm{s}}_{\textrm{LV}} \times (0, T), \end{aligned}$$

where $[\textrm{Ca}^{2+}]_{\textrm{i}}$ represents the intracellular calcium concentration stored in the vector function $\varvec{z}$. Following^55^, Eq. (3) allows then to compute the active contractile force $T_{\textrm{act}}(\varvec{s}, \textrm{SL})$.

For the structural problem, we consider the *elastodynamic equation*, in the unknown $\varvec{d}^{\textrm{s}}$, in which the first Piola-Kirchhoff stress tensor is split into a passive term (depending on $\varvec{d}^{\textrm{s}}$ only) and an active term (depending on $\varvec{d}^{\textrm{s}}$ and $T_{\textrm{act}}$). For the passive part, we use the *Usyk anistropic strain energy function*^57^. In short, we denote the structural problem as

4 $$\begin{aligned} {\mathscr {M}} (\varvec{d}^{\textrm{s}}; T_{\textrm{act}}, \varvec{p}_{\textrm{cavity}}) = 0 \; \qquad \text { in } \widehat{\Omega }^{\textrm{s}}\times (0, T), \end{aligned}$$

equipped with the following boundary conditions: generalized Robin boundary conditions^33^ to model the action of the pericardium, homogeneous Dirichlet boundary conditions (i.e. no displacement) on the rings of the pulmonary veins and homogenous Neumann boundary conditions (i.e. no stress) on the ring of the ascending aorta. Furthermore, for simplicity, we set homogenous Dirichlet boundary conditions on the epicardial valvular ring. On the endocardium, we set the fluid pressure as described in the following paragraph.

In this work, we consider a one-way coupling between the electromechanics and the 3D fluid dynamics–Darcy problems^58–61^ (see below). Specifically, the 3D electromechanics problem is solved prior to the 3D fluid dynamics-perfusion problem. However, in order to account for feedback of the fluid on the electromechanical model, we strongly couple the 3D electromechanics with a 0D lumped parameter model of the circulation^33,62,63^. This choice guarantees overall full consistency between mechanics and fluid dynamics, thanks to the 3D-0D coupling. Then, it allows for increased resolution of the fluid dynamics description near the regions of interest, while maintaining compatibility with the electromechanics problem Specifically, on the endocardium, we enforce the continuity of the 0D fluid-3D solid cavity pressures and cavity volumes. Accordingly, $\varvec{p}_{\textrm{cavity}}$ and $\varvec{V}_{\textrm{cavity}}$ are the vectors collecting the pressures and volumes of the left atrium, left ventricle and ascending aorta. We denote the circulation model as

5 $$\begin{aligned} {\mathscr {C}} (\varvec{c}, \varvec{V}_{\textrm{cavity}}^{\textrm{0D}}; \varvec{p}_{\textrm{cavity}}) = 0 \; \qquad \text { in } (0, T), \end{aligned}$$

where $\varvec{c}$ is the state vector that includes pressures, volumes and fluxes in different compartments. Particularly, the pressure $\varvec{p}_{\textrm{cavity}}$ acts as a Lagrangian multiplier to enforce the volumetric costraint $\varvec{V}_{\textrm{cavity}}(\varvec{d}^{\textrm{s}}) = \varvec{V}_{\textrm{cavity}}^{\textrm{0D}}$^33^.

#### The fluid geometry and fluid dynamics models

Let $\widehat{\Omega }^{\textrm{f}}\subset {\mathbb {R}}^3$ be the fluid dynamics domain (that is the region occupied by the fluid) in its reference configuration. The fluid domain in the current configuration is shown in Fig. 2 and defined as $\Omega ^{\textrm{f}}= \{ \varvec{x} \in {\mathbb {R}}^3: \; \varvec{x} = {\widehat{\varvec{x}}}+ \varvec{d}^{\textrm{f}}({\widehat{\varvec{x}}}, t), \; {\widehat{\varvec{x}}}\in \widehat{\Omega }^{\textrm{f}}\},$ with $\varvec{d}^{\textrm{f}}: \widehat{\Omega }^{\textrm{f}}\times (0, T)$ being the domain displacement (for the sake of brevity, we omit the subscript *t* from the fluid domain and its boundaries). The latter is computed by solving a Laplace equation in ${\widehat{\Omega }^{\textrm{f}}} \times (0, T)$ with Dirichlet boundary conditions on the physical wall: $\varvec{d}^{\textrm{f}}= \varvec{d}^{\textrm{f}}_{w}$ , with $\varvec{d}^{\textrm{f}}_{w}$ equal to the electromechanical displacement $\varvec{d}^{\textrm{s}}$ restricted on the endocardium. Furthermore, for simplicity, we set zero displacement on the coronaries walls. We make this assumption consistently with our choice of using a biventricular fixed domain. Furthermore, since the aorta is moving and the coronaries are fixed, artifacts at their interface can arise. Thus, we suitably smooth the displacement in the interface regions using methods and tools described in^61,64^. We compute the fluid domain velocity by $\varvec{u}^{\textrm{ALE}}= \frac{\partial \varvec{d}^{\textrm{f}}}{\partial t}$. We compactly denote the *fluid geometry problem* as

6 $$\begin{aligned} {\mathscr {G}}(\varvec{d}^{\textrm{f}}, \varvec{u}^{\textrm{ALE}}; \varvec{d}^{\partial \Omega }) = 0 \qquad \text { in } \widehat{\Omega }^{\textrm{f}}\times (0, T). \end{aligned}$$

To model blood flows in the left heart and large epicardial coronaries, we consider the *Navier–Stokes* equations expressed in *Arbitrary Lagrangian Eulerian* (ALE) framework^65^. We set our fluid dynamics problem in the domain $\Omega ^{\textrm{f}}$, delimited by $\partial \Omega ^{\textrm{f}}= \Gamma ^{\textrm{f}}_{\textrm{pv}} \cup \Gamma ^{\textrm{f}}_{\textrm{aa}} \cup \Gamma ^{\textrm{f}}_{\textrm{c}} \cup \Gamma ^{\textrm{f}}_{\textrm{w}}$. These boundaries represent the pulmonary veins sections, ascending aorta section, coronary outlet sections and endocardial wall, respectively (see Fig. 2, top). In particular, we consider *J* coronary outlet sections: $\Gamma ^{\textrm{f}}_{\textrm{c}} = \cup _{j=1}^J \Gamma _{\textrm{c}}^{\textrm{f}, j}$. We denote by $\varvec{u}^{\textrm{f}}$ and $p^{\textrm{f}}$ the fluid velocity and pressure, respectively. We model blood as if it were a Newtonian fluid with constant density $\rho = 1.06\cdot 10^{3}\,\hbox {kg}/\hbox {m}^{3}$ and dynamic viscosity $\mu =3.5\cdot 10^{-3}\,\hbox {kg}/(\hbox {ms})$. Moreover, we account for the presence of both mitral and aortic valve by means of the *Resistive Immersed Implicit Surface* (RIIS) method^66,67^. We refer to refs.^6,61,66,68–70^ for further details, extensions, and clinical applications of this method. We let the valves open and close instantaneously, at the initial and final times of isovolumetric phases (computed from the electromechanical simulation). The fluid dynamics model reads:

7 $$\begin{aligned} {\mathscr {F}}(\varvec{u}^{\textrm{f}}, \, p^{\textrm{f}}\, ; \varvec{u}^{\textrm{ALE}}, p_{\textrm{c}}^j) = 0 \qquad \text {in } \Omega ^{\textrm{f}}\times (0, T) \end{aligned}$$

At the wall, we prescribe the ALE velocity (computed in Eq. (6)). On the coronary outlets, we consider coupling conditions with the Darcy model (see below). Moreover, on $\Gamma ^{\textrm{f}}_{\textrm{c}}$, we also assume null tangential tractions^18^. Furthermore, on $\Gamma ^{\textrm{f}}_{\textrm{aa}}$ and $\Gamma ^{\textrm{f}}_{\textrm{pv}}$, we prescribe Neumann boundary conditions. Specifically, on the outlet section of the ascending aorta, we prescribe the systemic arterial pressure resulting from the 3D-0D electromechanical simulation. Moreover, we set a constant physiological pressure equal to 10 mmHg on the inlet pulmonary vein sections. Notice that we set a constant pressure value because the 0D circulation model provides the pressure in a compartment immediately after the lungs which does not correspond to the inlet left atrial pressure. The fluid dynamics model is also equipped with a zero velocity initial condition.

#### The multi-compartment Darcy model

To model blood perfusion, we consider a multi-compartment Darcy model in the biventricular myocardial domain $\Omega ^{\textrm{p}}$ (see Fig. 2, top). This model allows us to describe several length scales featuring the myocardium and its microvasculature as a porous medium made of different compartments^4,18,19,71^. Specifically, we consider the three compartments Darcy equations^18,19^ in the unknown $\varvec{u}^{\textrm{p}}_i$, $p^{\textrm{p}}_i$, representing the Darcy velocity and pore pressure, respectively, with $i=1, 2, 3$:

$$\begin{aligned} ({\mathscr {P}})\left\{ \begin{array}{lll} \varvec{u}^{\textrm{p}}_i + K_i \nabla p^{\textrm{p}}_i = \textbf{0} &{} \text { in } \Omega ^{\textrm{p}}\times (0, T), &{} \qquad \qquad (8\textrm{a}) \\ \nabla\cdot \varvec{u}^{\textrm{p}}_i = g_i - \mathop \sum \limits _{k=1}^3\beta _{i, k} (p^{\textrm{p}}_i - p^{\textrm{p}}_k) &{} \text { in } \Omega ^{\textrm{p}}\times (0, T), &{} \qquad \qquad (8\textrm{b})\\ \varvec{u}^{\textrm{p}}_i \cdot \varvec{n} = 0 &{} \text { on } \partial \Omega ^{\textrm{p}}\times (0, T). &{} \qquad \qquad (8\textrm{c}) \end{array}\right. \end{aligned}$$

$K_i$ is the permeability tensor, $g_i$ a volumetric sink term and the coefficients $\beta _{i, k}$ are the inter-compartment pressure-coupling coefficients. Following ref.^18^, $g_1$ is provided by epicardial blood hemodynamics (i.e. by the coupling condition with the Navier–Stokes problem, see below) and $g_2=0$ since the second compartment does not exchange mass with the outside. Furthermore, to account for the effect of the cardiac contraction on perfusion—still avoiding the use of a poromechanical model^20^—we propose $g_3$ to surrogate the reservoir effect of the coronary bed by making the following phenomenological assumption:

9 $$\begin{aligned} g_3=-\gamma (p^{\textrm{p}}_3-p_{\textrm{bed}}),\qquad p_{\textrm{bed}}(t) = a_1 p_{\textrm{LV}}(t) + a_2, \end{aligned}$$

where $\gamma$ is a suitable coefficient and the new contribution $p_{\textrm{bed}}(t)$ is a function of the left ventricular pressure $p_{\textrm{LV}}(t)$. The latter is obtained from the 3D-0D electromechanical problem (1), (2), (3), (4) and (5). Specifically, $p_{\textrm{LV}}(t)$ is stored into the vector $\varvec{p}_{\textrm{cavity}}$. In (9), $a_1$ and $a_2$ are calibrated using as a criterion the obtainment of physiological diastolic coronary flowrate.

The biventricular domain $\Omega ^{\textrm{p}}$ is partitioned into *J* non-overlapping perfusion regions, such that each epicardial vessel feeds only one portion^18^. For the estimation of parameters $K_i$, $\beta _{i, k}$, with $i, k = 1, 2, 3$, and for the strategy employed to partion $\Omega ^{\textrm{p}}$, we refer to ref.^18^.

#### Coupling conditions

In this section, we describe the coupling conditions enforced to match the different physics. In Fig. 2 bottom, we sketch the overall multiphysics model and we highlight the coupling conditions. For the fully coupled electromechanical model, we refer the reader entirely to ref.^33^.

For the coupling between electromechanics and cardiac hemodynamics, we consider the following kinematic condition:

10 $$\begin{aligned} \varvec{u}^{\textrm{f}}= \dot{\varvec{d}^{\textrm{s}}} \qquad \text { on } \Gamma _{\textrm{w}} \times (0, T), \end{aligned}$$

where $\varvec{d}^{\textrm{s}}$ is defined on the atrial and ventricular endocardium and on the endothelium of the ascending aorta. We recall that, for simplicity, we set null displacement on the coronaries wall. Furthermore, we highlight that, within the framework of the one-way coupling hypothesis, we have made the choice to prioritize neglecting the dynamic balance over the kinematic continuity between electromechanics and hemodynamics. Our selection is motivated by the fact that the fluid dynamics problem inherently relies on a displacement field to deform the cardiac chambers, which subsequently drives the flow of blood.

For the fully coupled Navier–Stokes–Darcy model, the coupling conditions read^18^:

11a $$\begin{aligned}{}&-\left( \nabla \varvec{u}^{\textrm{f}}+ \left( \nabla \varvec{u}^{\textrm{f}}\right) ^T \right) \varvec{n} \cdot \varvec{n} - p + \frac{1}{\alpha _j} \int _{\Gamma _{\textrm{c}}^{\textrm{f}, j}} \varvec{u}^{\textrm{f}}\cdot \varvec{n} = \frac{1}{|{\Omega ^{\textrm{p}}}^j|} \int _{{\Omega ^{\textrm{p}}}^j} p^{\textrm{p}}_1 \, \textrm{d}\varvec{x} \quad \text {on } \Gamma _{\textrm{c}}^{\textrm{f}, j} \times (0, T), \text { with } j = 1, \dots , J, \end{aligned}$$

11b $$\begin{aligned}{}&g_1 (\varvec{x}) = \sum _{j=1}^{J} \frac{\chi _{{\Omega ^{\textrm{p}}}^j} (\varvec{x})}{{|{\Omega ^{\textrm{p}}}^j|}} \int _{\Gamma _{\textrm{c}}^{\textrm{f}, j}} \varvec{u}^{\textrm{f}}\cdot \varvec{n} \quad \text {in } \Omega ^{\textrm{p}}\times (0, T). \end{aligned}$$

where Eqs. (11a) and  (11b) enforce the balance of internal forces and mass conservation, respectively. In (11a) $\alpha _j$ are the conductances. Moreover, in (11b), $\chi _{{\Omega ^{\textrm{p}}}^j}$ is the characteristic function of the *j*–th partition, with $j=1, \dots , J$^18^.

### Computational setup

**Figure 3:**
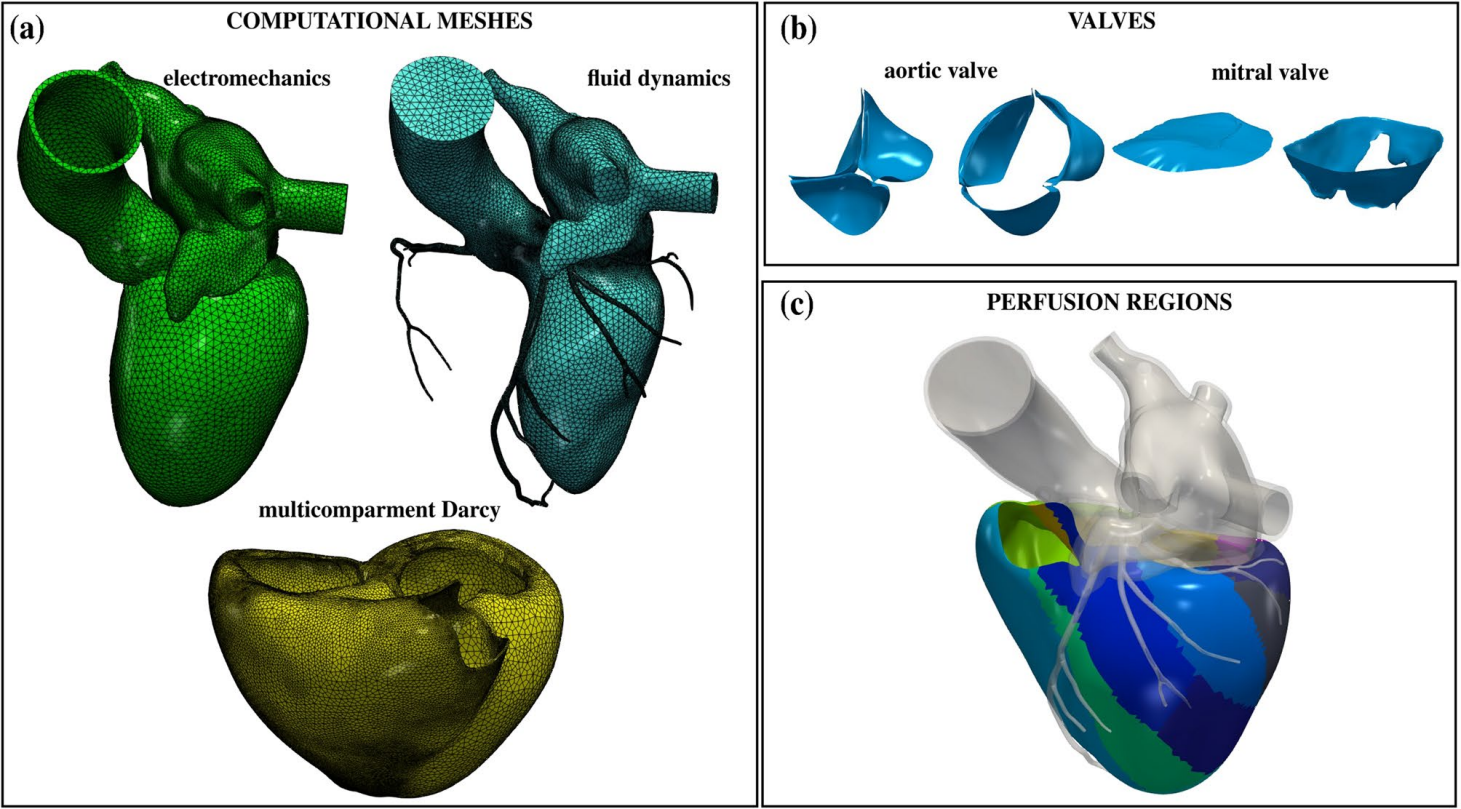
(**a**) The three computational meshes for the multiphysics problem. (**b**) Aortic and mitral valves in the open and closed configuration. (**c**) Perfusion regions in the biventricular geometry.

We consider a realistic cardiac geometry provided by the Zygote solid 3D heart model^72^: an anatomically CAD model representing an average healthy human heart reconstructed from high-resolution CT scan data. We generate three meshes for the electromechanics, fluid dynamics and multicompartment Darcy problems with vmtk^73^, using the methods and tools discussed in^64^. Details on the generated meshes are provided in Table 1 and displayed in Fig. 3a. Note that the CFD mesh is refined near the valves to accurately capture them with the RIIS method^6,61,66^. Immersed valves in their open and closed configurations are displayed in Fig. 3b. Notice also that we used the same mesh for electrophysiology and mechanics, with a value of the mesh size which is tipically considered too coarse to accurately resolve the traveling electrical front^49,74^. However, we suitably increase the conductivities to compensate for the use of a coarse electrophysiological mesh^32,75,76^.

In Fig. 3c, we report the perfusion regions of the biventricular geometry: one for each terminal vessel. For the complete setup of the multicompartment Darcy model, and for the preprocessing methods used to generate the perfusion regions, we refer the interested reader to ref.^18^. To surrogate the reservoir effect of the coronary bed (see Eq. (9)), we choose $a_1 = 0.4$ and $a_2 = 1500$ Pa, which corresponds to a coronary bed pressure in the range [14.2, 61.4] mmHg, and we set $\gamma = 1\cdot 10^{-4}$ 1/(Pa s), as in ref.^18^. For a complete list of the parameters used in our simulations, we refer to the Appendix. We discretize our mathematical models in space by the Finite Element (FE) method. We use linear FEs for electrophysiology and mechanics. The fluid dynamics problem is solved with linear FEs with VMS-LES stabilization^77,78^, acting also as a turbulence model to account for possible transition-to-turbulence effects^48^. The convective term is treated semi-implicitly. The multicompartment Darcy model, solved for the pressures, is discretized with linear FEs. As temporal advancing scheme, we use Backward Difference Formula (BDF), with the time-step sizes listed in Table 1. For additional details on numerics, we refer to refs.^18,33,61^ for the electromechanics, fluid dynamics, and perfusion models, respectively.

To efficiently solve the coupled problem, we first solve the electromechanical problem using a *Segregated-Intergrid-Staggered* method^33,34^. We pick the displacement on the fifth heart cycle—once the solution has approached a period limit cycle in terms of pressure and volume transients—and we use it as unidirectional input^61^ (*one-way*) for the fully coupled fluid dynamics - multicompartment Darcy problem. The electromechanical displacement is linearly projected onto the CFD wall mesh. To solve the fluid dynamics—multicompartment Darcy problem, we use an *implicit method* with an *iterative splitting strategy*, i.e. we subiterate discretizations of (7) and (8) until convergence^18^ (with a relaxation factor equal to 0.1 that we use to accelarate convergence). The variables shared between the two problems are those defined in Eqs. (11b) and  (11a). We start our simulation at the end of the filling phase. We simulate two heartbeats and we report the solution of the second cycle to cancel the influence of a non-physical null velocity initial condition.

We solve the multiphysics problem in life$^{{\texttt {x}}}$^79^, a high-performance C++ FE library developed within the iHEART project, mainly focused on cardiac simulations, and based on the deal.II finite element core^80–82^. The source code of the life$^{\texttt {x}}$ module for hemodynamics simulations, referred to as life$^{\texttt {x}}$-cfd, has been recently released^83,84^ (https://lifex.gitlab.io/). Numerical simulations are run in parallel on the GALILEO100 supercomputer (528 computing nodes each 2 x CPU Intel CascadeLake 8260, with 24 cores each, 2.4 GHz, 384GB RAM) at the CINECA supercomputing center, using 288 cores.

**Table 1:** Mesh details and time step sizes for the electromechanics and fluid dynamics–Darcy simulations.

Simulation	Physics	Mesh size [mm]			Cells	Vertices	DOFs	$\Delta t$ [s]
		Min	Avg	Max				
Electromechanics	Electrophysiology	0.93	2.7	4.8	142056	31988	224410	$1\cdot 10^{-4}$
	Force generation						31988	$1\cdot 10^{-3}$
	Mechanics						95964	$1\cdot 10^{-3}$
	Circulation	–	–	–	–	–	–	$1\cdot 10^{-3}$
Fluid dynamics-Darcy	Fluid dynamics	0.03	0.92	4.03	1740644	304411	1217644	$5\cdot 10^{-4}$
	Darcy	0.31	1.78	5.03	214484	267374	802122	$5\cdot 10^{-4}$

Electrophysiology, force generation and mechanics are solved on the same left heart mesh.

## Results

**Figure 4:**
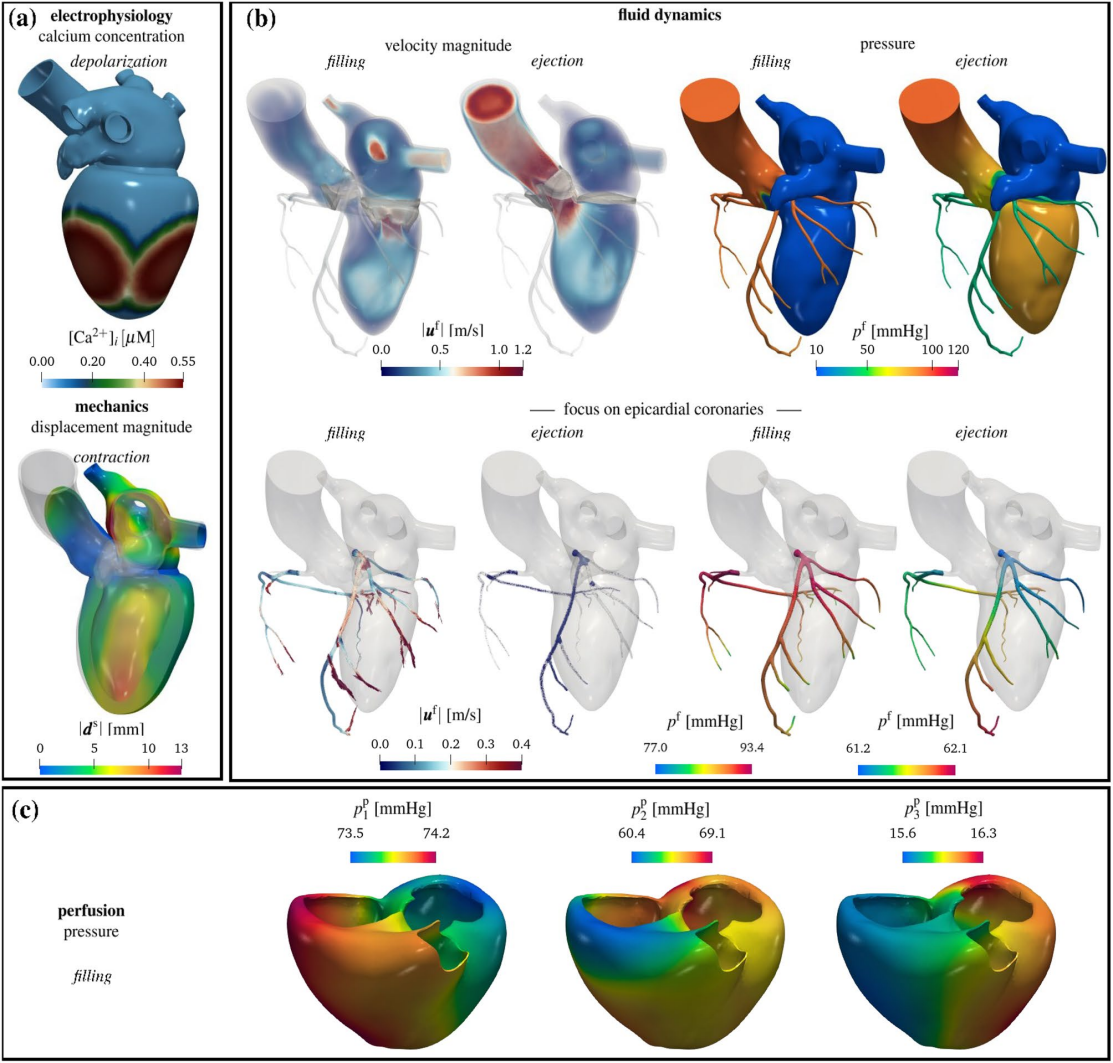
Results from a physiological simulation. (**a**) electromechanics of the left heart: calcium concentration during ventricular depolarization and displacement magnitude during ventricular contraction. (**b**) left heart hemodynamics: on the top, volume rendering of velocity magnitude and pressure during filling and ejection phases; on the bottom, focus on the epicardial coronary arteries. (**c**) myocardial perfusion: Darcy pressure in three different compartments during filling. Test I.

We start our analysis from a physiological simulation of a coupled electromechanics–blood dynamics–myocardial perfusion obtained by means of the proposed multiphysics model (Test I). In Fig. 4, we report results from this test. Concerning electromechanics (Fig. 4a), we show the calcium concentration, along with the displacement magnitude when the ventricle contracts. We display the intracardiac hemodynamics during filling and ejection in Fig. 4b, by reporting the volume rendering of velocity magnitude and pressure on the boundary of $\Omega ^{\textrm{f}}$. Notice that the model can faithfully predict the formation of the clockwise jet in the left ventricle during filling, which redirects the blood in the aortic root during systole^85,86^. Considering the cardiac chambers only, we find larger velocities during ejection, compared to the filling phase. Conversely, focusing on the coronaries only (Fig. 4b, bottom), we notice that blood is faster during the filling with respect to the ejection phase; accordingly, a larger pressure drop is also computed in the coronary tree during ventricular diastole, allowing the blood to accelerate and to perfuse the cardiac muscle. In Fig. 4c, we report the multicompartment Darcy’s pressures during the filling stage.

**Figure 5:**
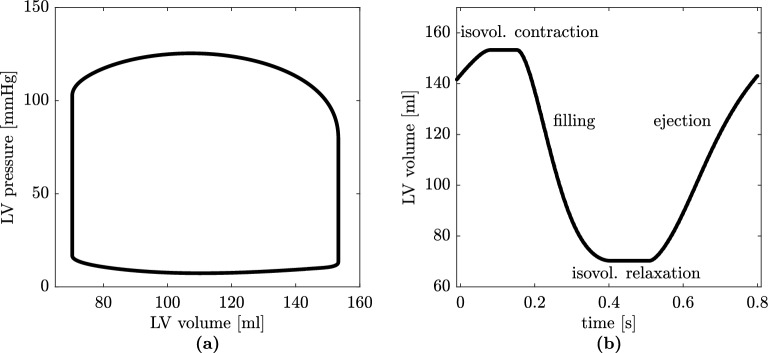
Results from physiological 3D electromechanical - 0D circulation simulation: (**a**) left ventricular pressure-volume loop; (**b**) left ventricular volume versus time. Test I.

Figure 5 shows quantitative results of the electromechanical simulation in Test I. The validation of the standalone electromechanical model in terms of several biomarkers have been carried out in^33,34^. Here we report some of these biomarkers, whose values are fundamental to assessing the physiological relevance of the overall model in terms of myocardial perfusion, since driven by electromechanics. Conversely, the CFD of the left heart fully coupled with the Darcy model of the biventricular geometry is a novel aspect of this study, making all the reported biomarkers unique contributions of this paper. Consistently with clinical ranges from literature^87–89^ we compute the left ventricular stroke volume, ejection fraction, and peak pressure (the latter coming from the 0D hemodynamic model) equal to 83.0 ml, 54.2%, and 125.4 mmHg, respectively (see Fig. 5a,b, where pressure-volume loop and volume in time are represented).

**Figure 6:**
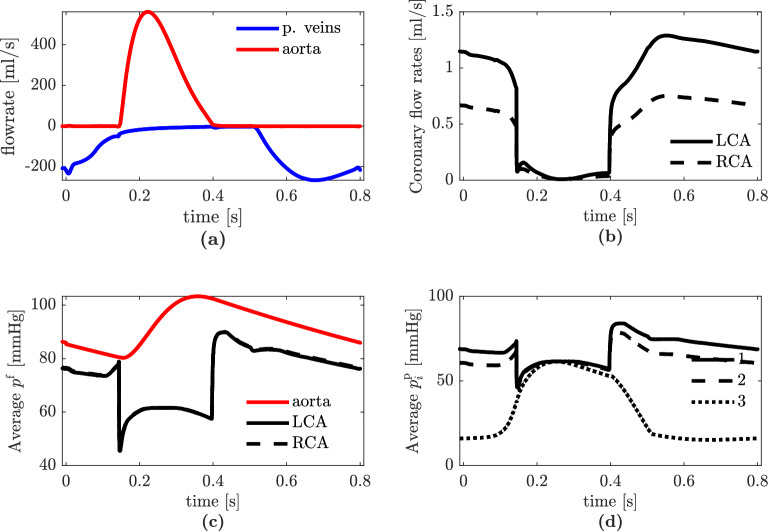
Results from physiological 3D Navier–Stokes–3D Darcy simulation in a representative heartbeat: (**a**) flow rates in pulmonary veins and aortic outlet section; (**b**) flow rates in epicardial coronary arteries; (**c**) average pressure in aortic outlet sections and coronary outlets; (**d**) pressure in the three Darcy’s compartments. Test I.

From Fig. 5b, it is possible to distinguish isovolumetric contraction, systolic ejection, isovolumetric relaxation, and diastolic filling phases. We show transients of the Navier–Stokes–Darcy simulation in Fig. 6. We report the flow rate computed at the aortic section and the total flow rate in the pulmonary veins in Fig. 6c: we compute a peak aortic flow equal to 562.0 ml/s—consistently with physiological ranges^90^—and the peak total flux in the pulmonary veins is 267.2 ml/s. In Fig. 6d, we show the total fluxes computed at the outlets of the Left Coronary Artery (LCA) and Right Coronary Artery (RCA). Our mathematical model faithfully predicts a peak blood flow rate at the beginning of the filling phase (diastole), resulting from the myocardium relaxation after the systolic contraction. Our finding is consistent with clinical evidences^91^; furthermore, as also experimentally measured in^92^, the flux in the LCA is larger than the one in the RCA. Pressures in the fluid dynamics domain are reported in Fig. 6e. We obtain a peak systolic arterial pressure of 103.3 mmHg and a minimum diastolic pressure equal to 80.3 mmHg: both results are physiologically consistent^93^. In Fig. 6e, we also show the coronary pressure by averaging the average pressure in each coronary outlet: we get similar LCA and RCA pressures. Figure 6f shows the pressure in the three Darcy’s compartments. As expected, we have a decreasing pressure going from one compartment to the following one, and comparable values during the systolic peak, due to the contraction of the muscle and the consequent partial obstruction of vessels.

**Figure 7:**
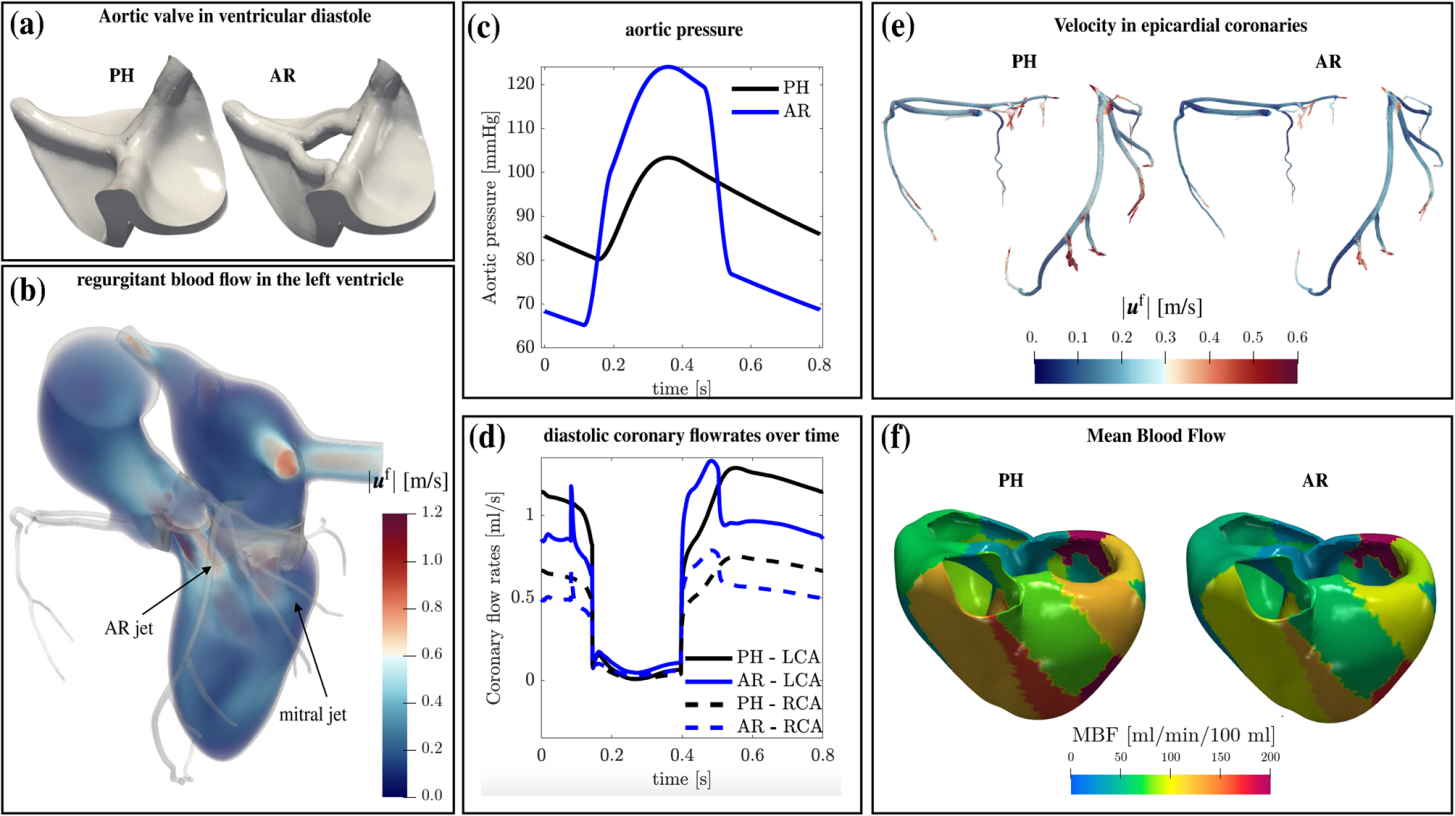
Simulation of aortic r egurgitation (AR): (**a**) aortic valve in ventricular diastole modeled with the RIIS method, comparison between physiological (PH) and AR cases; (**b**) volume rendering of velocity magnitude during ventricular filling; (**c**) aortic pressure prescribed on the ascending aorta outlet section in the PH and AR cases; (**d**) coronary flowrates over time, comparison between PH and AR cases; (**e**) velocity during diastole in the coronary tree, comparison between PH and AR cases; (**f**) Mean Blood Flow at diastolic peak in PH and AR cases. Test II and comparisons with PH case.

We aim now to study the effects that a valvular pathology has on myocardial perfusion. This allows us to explore the capabilities of the mathematical model in simulating also pathological scenarios. To this aim, we consider Test II, where the case of *Aortic Regurgitation* (AR) is considered. This pathology consists of a leaking of the aortic valve leaflets causing the blood to flow from the aorta to the left ventricle during the filling stage. To model the leaking of the aortic valve, we replace the “closed” physiological configuration PH used in Test I by the regurgitant configuration AR used in Test II, as we display in Fig. 7a. We obtain the AR configuration by introducing a regurgitant orifice which is about the 4.5% of the aortic annulus section. Furthermore, since AR is associated with an increased systolic and a decreased diastolic aortic pressure^94^, we modify the systemic arterial pressure prescribed on $\Gamma _{\textrm{aa}}^{\textrm{f}}$ accordingly. In fact, we increase and decrease the pressure by 20% in systole and diastole, respectively (see Fig. 7c). Figure 7b shows the volume rendering of the velocity magnitude in the AR case. During the filling stage, we observe reverse blood flow from the aorta to the left ventricle, yielding a mix of blood between the mitral and AR jets. In Fig. 7d, we compare the coronary flowrates against time in the PH and AR cases. The diastolic flowrate decreases in the AR case, with a peak reduction of 24.8%. This trend is also confirmed by Fig. 7e, where we show the velocity glyphs in the coronary tree at the diastolic peak: in the AR case, we measure much lower velocities. Differently, during ejection, we observe that the AR case produces a slight increase of the coronary flow (Fig. 7d) due to a larger systemic arterial pressure than in the PH case. To better assess the consequences of this pathology in terms of myocardial perfusion, we quantify the amount of blood inside the microvascolature. Accordingly, we compute the Myocardial Blood Flow (MBF) as^18,24^:

12 $$\begin{aligned} \textrm{MBF}(\varvec{x}) = \beta _{2, 3} (\varvec{x}) \; (p^{\textrm{p}}_2(\varvec{x}) - p^{\textrm{p}}_3(\varvec{x})) \; 60 [\hbox {s}/\hbox {min}] \cdot \; 100 [\hbox {mL}/100\hbox {g}]. \end{aligned}$$

MBF represents the amount of blood reaching the third compartment, i.e. where oxygen and nutrients are exchanged at a capillary level. This value is normalized over 100 g of cardiac tissue and the factor 60 s/min allows to express the perfusion rate in minutes. The unit used for the inter-compartment pressure-coupling coefficients is g/(s Pa mL), that for the pressure is Pa. As in ref.^18,24^, we have assumed the tissue to have unit density, so that the unit measure of MBF is  mL/min/100mL, accordingly with stress-CTP technology^95^. Figure 7f shows a comparison between the PH and AR cases in terms of MBF at the diastolic peak. Overall, in both cases, we can observe a heterogeneous distribution of the MBF due to different resistance of the vessels inside each perfusion region, provided by the heterogeneous parameters of the Darcy model^18^. More quantitatively, in the PH case, we compute an average MBF equal to 87.5 mL/min/100mL. Our result is consistent with clinical studies, which measure a normal MBF at rest from 57.6 to 96.1 mL/min/100mL^96^. Differently, the pathological case is characterized by a much lower perfusion: at the diastolic peak we measure 68.2 mL/min/100mL. Thus, the ventricular reverse flow due to a regurgitant aortic valve is responsible for a steal of coronary flow, and hence an abnormal and impaired myocardial perfusion.

## Discussion

This paper introduces a new computational model that integrates multiple cardiac physical processes, including electrophysiology, active and passive mechanics, blood dynamics, and myocardial perfusion, within a comprehensive 3D representation. The distinctive feature of this study is the systematic coupling of blood flow from the left atrium to the epicardial coronary vessels, all within a unified geometry. We successfully applied the model to both healthy and an aortic regurgitant scenarios. By carrying out simulations on a realistic cardiac geometry, we showed that the model faithfully predicted electromechanics and blood dynamics quantities as previously shown also in^33,56,61^. Moreover, as a new outcome of this work, we were able to predict cardiac perfusion in both physiological and aortic regurgitant cases by means of a comprehensive cardiac function model.

The inclusion of the whole left heart function and geometry in our simulations allowed us to avoid any arbitrary prescription of the fluid pressure and velocities at the inlet of epicardial coronaries. Indeed, in previous perfusion models, due to the absence of any electromechanical and fluid dynamics model in the left ventricle, it was necessary to prescribe transients of flowrates or pressures at such sections^18,21–24^. Moreover, it is well known that vascular resistance increases during systole because the contraction of the myocardium compresses the intramyocardial coronary arteries^7^, producing a peak flowrate during diastole. In our simulations, we can correctly capture this phenomenon without prescribing any data on the inlet of coronaries, but thanks to the interplay of different features that we included in the model, as discuss in what follows.

- By including the entire left heart geometry and modeling its motion through the use of an electromechanics model, we were able to achieve a physiological blood velocity pattern in the ascending aorta.
- By modeling the aortic valve during the ejection phase, we were able to simulate partial obstruction of the coronary ostia when the valve is open. This approach resulted in a reduction of the systolic coronary flow rate, which is consistent with clinical evidence^97^. Our simulations indicated that neglecting the modeling of the aortic valve in its open configuration (i.e. by setting a null resistance during systole) leads to the computation of larger and non-physiological flow rates. For the sake of brevity, we do not include the results of this case.
- The contraction of cardiomyocytes is a well-known cause of impediment to systolic coronary flow^98,99^. To account for this effect in our perfusion model, we made a phenomenological assumption on the coronary bed pressure by introducing a novel time-dependent pressure (refer to Eq. 9) that emulates an increase in vascular resistance^97^, thereby enabling the simulation of a diastolic coronary flow rate.

To test the physiological relevance of our computational model, we compared several biomarkers obtained by our computation against clinical ranges coming from the literature. Particularly, we computed the blood flow maps and the corresponding average MBF, getting results in accordance with the normal ranges.

Furthermore, the development of a multiphysics mathematical model allowed us to investigate that a regurgitant aortic valve produces a reduction of coronary flow during diastole, by redirecting the blood in the left ventricle, as highlighted in Fig. 7b,d. The main consequence of this aspect is a reduced perfusion of the myocardium during diastole, accordingly with clinical evidence^100^ and quantified by the computation of a reduced MBF at the diastolic peak (see Fig. 7f).

Moreover, we faithfully captured also a slight increase of the epicardial coronary flow during ejection with respect to the physiological case, as described in^100,101^ (see Fig. 7d, blue lines during ejection). This is due to a larger aortic pressure during systole, with respect to the physiological case.

The study presented in this work features some limitations. We highlight that setting a null displacement on the epicardial valvular ring in the electromechanical simulation is not physiological. Indeed, the base of the ventricle should be free to move up and down during the cardiac cycle. Accordingly, the coronaries should follow this motion. Moreover, they should be compliant, whereas we have assumed here that they are rigid and static. Furthermore, differently from electromechanics and fluid dynamics, we consider the multicompartment Darcy model in a fixed domain, as commonly done in the literature^16,18,24^. Although this represents a simplification of the model, we believe that even considering a moving domain for the Darcy model, our results for instance in terms of blood flow maps would not change significantly. In addition, a higher resolution mesh would be needed for electrophysiology to better capture the traveling wavefront and, consequently, to better describe the active ventricular contraction.

We noticed that the coronary pressure during ejection found by our simulation in subject PH is smaller if compared with standard physiological values and other computational studies^8,102^. In particular, from Fig. 6c, the LCA and RCA pressures should be about 25 mmHg greater during ejection. We believe that this is due to the open aortic valve configuration which is representative and not obtained by an FSI simulation. This limitation may result in an excessive occlusion of the coronary ostia and then in an augmented resistance during systole, which provokes a decrease of the computed coronary pressure.

In conclusion, we expect that the incorporation of the feedback between cardiac hemodynamics and tissue mechanics through the development of a fully coupled electro-mechano-fluid-perfusion model would enable the simulation and modeling of additional pathological scenarios, such as myocardial ischemia resulting from coronary artery occlusion. We believe that the present work represents an important milestone toward the achievement of this goal.

### Supplementary Information

Supplementary Information.

## Data Availability

The datasets used and/or analysed during the current study are available from the corresponding author on reasonable request.

## References

[1] Spaan J (2008). Coronary structure and perfusion in health and disease. Philos. Trans. R. Soc. A Math. Phys. Eng. Sci..

[2] Vankan WJ (1997). Finite-element simulation of blood perfusion in muscle tissue during compression and sustained contraction. Am. J. Physiol. Heart Circ. Physiol..

[3] Vankana W (1998). Mechanical blood-tissue interaction in contracting muscles: A model study. J. Biomech..

[4] Huyghe JM, Van Campen DH (1995). Finite deformation theory of hierarchically arranged porous solids–i. Balance of mass and momentum. Int. J. Eng. Sci..

[5] Guerciotti B (2017). A computational fluid-structure interaction analysis of coronary y-grafts. Med. Eng. Phys..

[6] Fumagalli I (2020). An image-based computational hemodynamics study of the systolic anterior motion of the mitral valve. Comput. Biol. Med..

[7] Lee J, Smith NP (2012). The multi-scale modelling of coronary blood flow. Ann. Biomed. Eng..

[8] Kim HJ (2010). Patient-specific modeling of blood flow and pressure in human coronary arteries. Ann. Biomed. Eng..

[9] Sankaran S (2012). Patient-specific multiscale modeling of blood flow for coronary artery bypass graft surgery. Ann. Biomed. Eng..

[10] Kung E, Kahn AM, Burns JC, Marsden A (2014). In vitro validation of patient-specific hemodynamic simulations in coronary aneurysms caused by Kawasaki disease. Cardiovasc. Eng. Technol..

[11] Sengupta D (2012). Image-based modeling of hemodynamics in coronary artery aneurysms caused by Kawasaki disease. Biomech. Model. Mechanobiol..

[12] Schwarz EL, Pegolotti L, Pfaller MR, Marsden AL (2023). Beyond CFD: Emerging methodologies for predictive simulation in cardiovascular health and disease. Biophys. Rev..

[13] Papamanolis L (2021). Myocardial perfusion simulation for coronary artery disease: A coupled patient-specific multiscale model. Ann. Biomed. Eng..

[14] Smith N, Pullan A, Hunter PJ (2002). An anatomically based model of transient coronary blood flow in the heart. SIAM J. Appl. Math..

[15] Formaggia L, Lamponi D, Quarteroni A (2003). One-dimensional models for blood flow in arteries. J. Eng. Math..

[16] Chabiniok R (2016). Multiphysics and multiscale modelling, data-model fusion and integration of organ physiology in the clinic: Ventricular cardiac mechanics. Interface Focus.

[17] Michler C (2013). A computationally efficient framework for the simulation of cardiac perfusion using a multi-compartment Darcy porous-media flow model. Int. J. Numer. Methods Biomed. Eng..

[18] Di Gregorio S (2021). A computational model applied to myocardial perfusion in the human heart: From large coronaries to microvasculature. J. Comput. Phys..

[19] Hyde ER (2014). Multi-scale parameterisation of a myocardial perfusion model using whole-organ arterial networks. Ann. Biomed. Eng..

[20] Barnafi Wittwer NA (2022). A multiscale poromechanics model integrating myocardial perfusion and the epicardial coronary vessels. SIAM J. Appl. Math..

[21] Sun Z, Xu L (2014). Computational fluid dynamics in coronary artery disease. Comput. Med. Imaging Graph..

[22] Zhong L (2018). Application of patient-specific computational fluid dynamics in coronary and intra-cardiac flow simulations: Challenges and opportunities. Front. Physiol..

[23] Athani A (2021). Two-phase non-Newtonian pulsatile blood flow simulations in a rigid and flexible patient-specific left coronary artery (LCA) exhibiting multi-stenosis. Appl. Sci..

[24] Di Gregorio S (2022). Prediction of myocardial blood flow under stress conditions by means of a computational model. Eur. J. Nucl. Med. Mol. Imag..

[25] Chapelle, D. *et al.* Numerical simulation of the electromechanical activity of the heart. In *Proc. International Conference on Functional Imaging and Modeling of the Heart*, 357–365 (Springer, 2009).

[26] Marx L (2020). Personalization of electro-mechanical models of the pressure-overloaded left ventricle: Fitting of windkessel-type afterload models. Philos. Trans. R. Soc. A.

[27] Gurev V, Lee T, Constantino J, Arevalo H, Trayanova NA (2011). Models of cardiac electromechanics based on individual hearts imaging data. Biomech. Model. Mechanobiol..

[28] Trayanova NA, Constantino J, Gurev V (2011). Electromechanical models of the ventricles. Am. J. Physiol. Heart Circ. Physiol..

[29] Dal H, Göktepe S, Kaliske M, Kuhl E (2013). A fully implicit finite element method for bidomain models of cardiac electromechanics. Comput. Methods Appl. Mech. Eng..

[30] Lafortune P, Arís R, Vázquez M, Houzeaux G (2012). Coupled electromechanical model of the heart: Parallel finite element formulation. Int. J. Numer. Methods Biomed. Eng..

[31] Augustin CM (2016). Anatomically accurate high resolution modeling of human whole heart electromechanics: A strongly scalable algebraic multigrid solver method for nonlinear deformation. J. Comput. Phys..

[32] Gerach T (2021). Electro-mechanical whole-heart digital twins: A fully coupled multi-physics approach. Mathematics.

[33] Regazzoni F (2022). A cardiac electromechanical model coupled with a lumped-parameter model for closed-loop blood circulation. J. Comput. Phys..

[34] Fedele M (2023). A comprehensive and biophysically detailed computational model of the whole human heart electromechanics. Comput. Methods Appl. Mech. Eng..

[35] Raghavan M, Ma B, Fillinger M (2006). Non-invasive determination of zero-pressure geometry of arterial aneurysms. Ann. Biomed. Eng..

[36] Sellier M (2011). An iterative method for the inverse elasto-static problem. J. Fluids Struct..

[37] Bols J (2013). A computational method to assess the in vivo stresses and unloaded configuration of patient-specific blood vessels. J. Comput. Appl. Math..

[38] Bayer JD, Beaumont J, Krol A (2005). Laplace-Dirichlet energy field specification for deformable models. An fem approach to active contour fitting. Ann. Biomed. Eng..

[39] Doste R (2019). A rule-based method to model myocardial fiber orientation in cardiac biventricular geometries with outflow tracts. Int. J. Numer. Methods Biomed. Eng..

[40] Bayer JD, Blake RC, Plank G, Trayanova NA (2012). A novel rule-based algorithm for assigning myocardial fiber orientation to computational heart models. Ann. Biomed. Eng..

[41] Piersanti R (2021). Modeling cardiac muscle fibers in ventricular and atrial electrophysiology simulations. Comput. Methods Appl. Mech. Eng..

[42] Fritz T, Wieners C, Seemann G, Steen H, Dössel O (2014). Simulation of the contraction of the ventricles in a human heart model including atria and pericardium. Biomech. Model. Mechanobiol..

[43] Pfaller MR (2019). The importance of the pericardium for cardiac biomechanics: From physiology to computational modeling. Biomech. Model. Mechanobiol..

[44] Strocchi M (2020). Simulating ventricular systolic motion in a four-chamber heart model with spatially varying robin boundary conditions to model the effect of the pericardium. J. Biomech..

[45] Santiago A (2018). Fully coupled fluid-electro-mechanical model of the human heart for supercomputers. Int. J. Numer. Methods Biomed. Eng..

[46] Bucelli M (2022). A mathematical model that integrates cardiac electrophysiology, mechanics and fluid dynamics: Application to the human left heart. Int. J. Numer. Methods Biomed. Eng..

[47] Sugiura S (2012). Multi-scale simulations of cardiac electrophysiology and mechanics using the university of Tokyo heart simulator. Prog. Biophys. Mol. Biol..

[48] Zingaro A, Dede’ L, Menghini F, Quarteroni A (2021). Hemodynamics of the heart’s left atrium based on a variational multiscale-les numerical method. Eur. J. Mech. B Fluids.

[49] Franzone PC, Pavarino LF, Scacchi S (2014). Mathematical Cardiac Electrophysiology.

[50] Salvador M (2022). The role of mechano-electric feedbacks and hemodynamic coupling in scar-related ventricular tachycardia. Comput. Biol. Med..

[51] Colli Franzone P, Pavarino L, Scacchi S (2017). Effects of mechanical feedback on the stability of cardiac scroll waves: A bidomain electro-mechanical simulation study. Chaos Interdiscip. J. Nonlinear Sci..

[52] Taggart P, Sutton PM (1999). Cardiac mechano-electric feedback in man: Clinical relevance. Prog. Biophys. Mol. Biol..

[53] Ten Tusscher KH, Panfilov AV (2006). Alternans and spiral breakup in a human ventricular tissue model. Am. J. Physiol. Heart Circ. Physiol..

[54] Ambrosi D, Pezzuto S (2012). Active stress vs. active strain in mechanobiology: Constitutive issues. J. Elast..

[55] Regazzoni F, Dedè L, Quarteroni A (2020). Biophysically detailed mathematical models of multiscale cardiac active mechanics. PLoS Comput. Biol..

[56] Zingaro, A. *et al.* An electromechanics-driven fluid dynamics model for the simulation of the whole human heart. *arXiv preprint* ArXiv:2301.02148 (2023).

[57] Usyk TP, LeGrice IJ, McCulloch AD (2002). Computational model of three-dimensional cardiac electromechanics. Comput. Vis. Sci..

[58] Augustin CM (2016). Patient-specific modeling of left ventricular electromechanics as a driver for haemodynamic analysis. EP Eur..

[59] Karabelas E (2018). Towards a computational framework for modeling the impact of aortic coarctations upon left ventricular load. Front. Physiol..

[60] Thilak A, Boilevin-Kayl L, Fernández MA, Gerbeau J-F (2020). Augmented resistive immersed surfaces valve model for the simulation of cardiac hemodynamics with isovolumetric phases. Int. J. Numer. Methods Biomed. Eng..

[61] Zingaro A (2022). A geometric multiscale model for the numerical simulation of blood flow in the human left heart. Discret. Contin. Dyn. Syst..

[62] Blanco PJ, Feijóo RA (2010). A 3d–1d-0d computational model for the entire cardiovascular system. Mecánica Comput..

[63] Hirschvogel M, Bassilious M, Jagschies L, Wildhirt SM, Gee MW (2017). A monolithic 3d–0d coupled closed-loop model of the heart and the vascular system: Experiment-based parameter estimation for patient-specific cardiac mechanics. Int. J. Numer. Methods Biomed. Eng..

[64] Fedele M, Quarteroni AM (2021). Polygonal surface processing and mesh generation tools for numerical simulations of the complete cardiac function. Int. J. Numer. Methods Biomed. Eng..

[65] Donea J, Giuliani S, Halleux J-P (1982). An arbitrary Lagrangian-Eulerian finite element method for transient dynamic fluid-structure interactions. Comput. Methods Appl. Mech. Eng..

[66] Fedele M, Faggiano E, Dede’ L, Quarteroni A (2017). A patient-specific aortic valve model based on moving resistive immersed implicit surfaces. Biomech. Model. Mechanobiol..

[67] Astorino M, Hamers J, Shadden SC, Gerbeau J-F (2012). A robust and efficient valve model based on resistive immersed surfaces. Int. J. Numer. Methods Biomed. Eng..

[68] Zingaro, A., Bucelli, M., Fumagalli, I., Dede’, L. & Quarteroni, A. Modeling isovolumetric phases in cardiac flows by an augmented resistive immersed implicit surface method. *Int J Numer Meth Biomed Engng.* e3767, (2023). 10.1002/cnm.3767 37615375

[69] Bennati L (2023). An image-based computational fluid dynamics study of mitral regurgitation in presence of prolapse. Cardiovasc. Eng. Technol..

[70] Bennati, L. *et al.* Turbulence and blood washout in presence of mitral regurgitation: A computational fluid-dynamics study in the complete left heart. *bioRxiv* 2023–03 (2023).

[71] Hyde ER (2013). Parameterisation of multi-scale continuum perfusion models from discrete vascular networks. Med. Biol. Eng. Comput..

[72] Zygote Media Group Inc, Zygote solid 3D heart generation II developement report. Technical Report (2014).

[73] Antiga L (2008). An image-based modeling framework for patient-specific computational hemodynamics. Med. Biol. Eng. Comput..

[74] Quarteroni A, Dede’ L, Manzoni A, Vergara C (2019). Mathematical Modelling of the Human Cardiovascular System: Data, Numerical Approximation, Clinical Applications.

[75] Pezzuto S, Hake J, Sundnes J (2016). Space-discretization error analysis and stabilization schemes for conduction velocity in cardiac electrophysiology. Int. J. Numer. Methods Biomed. Eng..

[76] Woodworth LA, Cansız B, Kaliske M (2021). A numerical study on the effects of spatial and temporal discretization in cardiac electrophysiology. Int. J. Numer. Methods Biomed. Eng..

[77] Forti D, Dedè L (2015). Semi-implicit BDF time discretization of the Navier-Stokes equations with VMS-LES modeling in a high performance computing framework. Comput. Fluids.

[78] Takizawa K (2014). ST and ALE-VMS methods for patient-specific cardiovascular fluid mechanics modeling. Math. Models Methods Appl. Sci..

[79] Africa PC (2022). lifex: A flexible, high performance library for the numerical solution of complex finite element problems. SoftwareX.

[80] Arndt D (2021). The deal. II library, version 9.3. J. Numer. Math..

[81] Arndt D (2020). The deal.II finite element library: Design, features, and insights. Comput. Math. Appl..

[82] deal.ii - an open source finite element library, accessed August 2023, https://www.dealii.org/.

[83] Africa, P. C. *et al.* lifex-cfd: an open-source computational fluid dynamics solver for cardiovascular applications. *arXiv preprint* arXiv:2304.12032 (2023).

[84] Africa, P. C., Fumagalli, I., Bucelli, M. & Zingaro, A. Lifex-CFD: An open-source computational fluid dynamics solver for cardiovascular applications. 10.5281/zenodo.7852089.

[85] Di Labbio G, Kadem L (2018). Jet collisions and vortex reversal in the human left ventricle. J. Biomech..

[86] Kilner PJ (2000). Asymmetric redirection of flow through the heart. Nature.

[87] Maceira AM, Prasad SK, Khan M, Pennell DJ (2006). Normalized left ventricular systolic and diastolic function by steady state free precession cardiovascular magnetic resonance. J. Cardiovasc. Magn. Reson..

[88] Clay S, Alfakih K, Radjenovic A, Jones T, Ridgway JP (2006). Normal range of human left ventricular volumes and mass using steady state free precession MRI in the radial long axis orientation. Magn. Reson. Mater. Phys., Biol. Med..

[89] Sugimoto T (2017). Echocardiographic reference ranges for normal left ventricular 2d strain: Results from the EACVI NORRE study. Eur. Heart J. Cardiovas. Imag..

[90] Hammermeister K, Brooks R, Warbasse J (1974). The rate of change of left ventricular volume in man: I. Validation and peak systolic ejection rate in health and disease. Circulation.

[91] Johnson K, Sharma P, Oshinski J (2008). Coronary artery flow measurement using navigator echo gated phase contrast magnetic resonance velocity mapping at 3.0 t. J. Biomech..

[92] Schiemann M (2006). Mr-based coronary artery blood velocity measurements in patients without coronary artery disease. Eur. Radiol..

[93] Hall JE, Hall ME (2020). Guyton and Hall Textbook of Medical Physiology.

[94] Maurer G (2006). Aortic regurgitation. Heart.

[95] Pontone G (2019). Dynamic stress computed tomography perfusion with a whole-heart coverage scanner in addition to coronary computed tomography angiography and fractional flow reserve computed tomography derived. JACC Cardiovasc. Imag..

[96] Kajander SA (2011). Clinical value of absolute quantification of myocardial perfusion with 15o-water in coronary artery disease. Circ. Cardiovasc. Imag..

[97] Padula RT, Camishion RC, Bollinger WF (1965). Obstruction of the coronary ostia during systole by the aortic valve leaflets. J. Thorac. Cardiovasc. Surg..

[98] Katz AM (2010). Physiology of the Heart.

[99] Vlachopoulos C, O’Rourke M, Nichols WW (2011). McDonald’s Blood Flow in Arteries: Theoretical, Experimental and Clinical Principles.

[100] Rabkin SW (2013). Differences in coronary blood flow in aortic regurgitation and systemic arterial hypertension have implications for diastolic blood pressure targets: A systematic review and meta-analysis. Clin. Cardiol..

[101] Kume T (2008). Mechanism of increasing systolic coronary flow velocity in patients with aortic regurgitation. J. Heart Valve Dis..

[102] Taylor CA, Fonte TA, Min JK (2013). Computational fluid dynamics applied to cardiac computed tomography for noninvasive quantification of fractional flow reserve: scientific basis. J. Am. Coll. Cardiol..

