# Supplementary material for: A comprehensive mathematical model for cardiac perfusion
Source: Sci Rep. 2023 Aug 30;13:14220. doi: 10.1038/s41598-023-41312-0 (PMC10469210; doi:10.1038/s41598-023-41312-0)
Supplement: Supplementary file 1 — Supplementary Information. [file 41598_2023_41312_MOESM1_ESM.pdf]

## Appendix

We report all the parameters we used to carry out the simulations. In Table 2, we list the parameters we used for electrophysiology, active force generation and mechanics. Table 3 contains the parameters used in the 0D circulation model. Details on the parameters used in the CFD model (in particular RIIS model) are provided in Table 4. Parameters of the perfusion model are given in Table 5 and Table 6.

| Physics | Parameter | | Value | |
|---|---|---|---|---|
| Electrophysiology (LV only) | Conductivities | $\sigma_m^l$ | $2.00 \cdot 10^{-4}$ | $m^2/s$ |
| | | $\sigma_m^t$ | $1.05 \cdot 10^{-4}$ | $m^2/s$ |
| | | $\sigma_m^n$ | $0.55 \cdot 10^{-4}$ | $m^2/s$ |
| | Stimulus | $A_{app}$ | 25.71 | V/s |
| | | $\sigma_{app}$ | $2.5 \cdot 10^{-3}$ | m |
| | | $T_{app}$ | $3 \cdot 10^{-3}$ | s |
| Active Force (LV only) | | $\gamma$ | 30 | |
| | | $k_d$ | 0.36 | |
| | | $\alpha_{k_d}$ | −0.2083 | |
| | | $K_{off}$ | 8 | 1/s |
| | | $K_{basic}$ | 4 | 1/s |
| | | $\mu_{fp}^0$ | 32.255 | 1/s |
| | | $\mu_{fp}^1$ | 0.768 | 1/s |
| | | $a_{XB}$ | $20 \cdot 10^8$ | Pa |
| Mechanics | Guccione | $\rho_s$ | 1000 | $kg/m^2$ |
| | | $c$ | $8.8 \cdot 10^2$ | Pa |
| | | $a_{ff}$ | 8 | |
| | | $a_{ss}$ | 6 | |
| | | $a_{nn}$ | 3 | |
| | | $a_{fs}$ | 12 | |
| | | $a_{fn}$ | 3 | |
| | | $a_{sn}$ | 3 | |
| | | $\kappa$ | $5 \cdot 10^4$ | Pa |
| | Boundary conditions | $K_\perp^{epi}$ | $2 \cdot 10^5$ | Pa/m |
| | | $K_\parallel^{epi}$ | $2 \cdot 10^4$ | Pa/m |
| | | $C_\perp^{epi}$ | $2 \cdot 10^4$ | Pa s/m |
| | | $C_\parallel^{epi}$ | $2 \cdot 10^3$ | Pa s/m |
| | In. conditions | $p_0$ | 1333.2 | Pa |

**Table 2.** Parameters used in the electromechanical model: electrophysiology, active force generation, and solid mechanics. For the force generation model, we only report parameters that are different from the original setting described in[55]. For a description of each parameter, we refer the reader to[33], where also the same notation is employed.

| | Parameter | Value | |
|---|---|---|---|
| Systemic arteries | $R_{AR}^{SYS}$ | 0.60 | mmHg s/mL |
| | $C_{AR}^{SYS}$ | 2.55 | mL/mmHg |
| | $L_{AR}^{SYS}$ | $2.7 \cdot 10^{-3}$ | mmHg s$^2$/mL |
| | $R_{upstream}^{SYS}$ | 0.05 | mmHg s/mL |
| | $p_{AR}^{SYS}(0)$ | 80.0 | Pa |
| | $Q_{AR}^{SYS}(0)$ | 0.0 | mL/s |
| Systemic veins | $R_{VEN}^{SYS}$ | 0.26 | mmHg s/mL |
| | $C_{VEN}^{SYS}$ | 60.0 | mL/mmHg |
| | $L_{VEN}^{SYS}$ | $5 \cdot 10^{-4}$ | mmHg s$^2$/mL |
| | $p_{VEN}^{SYS}(0)$ | 30.9 | Pa |
| | $Q_{VEN}^{SYS}(0)$ | 0.0 | mL/s |
| Pulmonary arteries | $R_{AR}^{PUL}$ | 0.05 | mmHg s/mL |
| | $C_{AR}^{PUL}$ | 10.0 | mL/mmHg |
| | $L_{AR}^{PUL}$ | $5 \cdot 10^{-4}$ | mmHg s$^2$/mL |
| | $p_{AR}^{PUL}(0)$ | 29.34 | Pa |
| | $Q_{AR}^{PUL}(0)$ | 0.0 | mL/s |
| Pulmonary veins | $R_{VEN}^{PUL}$ | 0.025 | mmHg s/mL |
| | $C_{VEN}^{PUL}$ | 38.4 | mL/mmHg |
| | $L_{VEN}^{PUL}$ | $2.083 \cdot 10^{-4}$ | mmHg s$^2$/mL |
| | $p_{VEN}^{PUL}(0)$ | 13.58 | Pa |
| | $Q_{VEN}^{PUL}(0)$ | 0.0 | mL/s |
| Right atrium | $E_A$ | 0.06 | mmHg/mL |
| | $E_B$ | 0.07 | mmHg/mL |
| | $t_C$ | 0.80 | |
| | $T_C$ | 0.17 | |
| | $T_R$ | 0.17 | |
| | $V_{RA}(0)$ | 64.17 | mL |
| Right ventricle | $E_A$ | 0.55 | mmHg/mL |
| | $E_B$ | 0.05 | mmHg/mL |
| | $t_C$ | 0.0 | |
| | $T_C$ | 0.34 | |
| | $T_R$ | 0.15 | |
| | $V_{RV}(0)$ | 148.9 | mL |
| Mitral valve | $R_{min}$ | 0.0075 | mmHg s/mL |
| | $R_{max}$ | 75 006.2 | mmHg s/mL |
| Aortic valve | $R_{min}$ | 0.0075 | mmHg s/mL |
| | $R_{max}$ | 75 006.2 | mmHg s/mL |
| Tricuspid valve | $R_{min}$ | 0.0075 | mmHg s/mL |
| | $R_{max}$ | 75 006.2 | mmHg s/mL |
| Pulmonary valve | $R_{min}$ | 0.0075 | mmHg s/mL |
| | $R_{max}$ | 75 006.2 | mmHg s/mL |

**Table 3.** Parameters of the circulation model for the electromechanical simulation. We refer to[33] for a description of each parameter, where the same notation is also employed.

| Physics | Parameter | | Value | |
|---|---|---|---|---|
| Fluid dynamics | | $\rho$ | $1.06 \cdot 10^3$ | kg/m$^3$ |
| | | $\mu$ | $3.5 \cdot 10^{-3}$ | kg/(m s) |
| | RIIS | $R_{MV}$ | $1 \cdot 10^4$ | kg/(m s) |
| | | $R_{AV}$ | $1 \cdot 10^4$ | kg/(m s) |
| | | $\varepsilon_{MV}$ | $2 \cdot 10^{-3}$ | mm |
| | | $\varepsilon_{AV}$ | $2 \cdot 10^{-3}$ | mm |

**Table 4.** Parameters used in the fluid dynamics model. For a description of the RIIS parameters, we refer the reader to[66], where the same notation is also used.

| Region | $K_1$ m$^2$/(Pa s) | $K_2$ m$^2$/(Pa s) | $K_3$ m$^2$/(Pa s) |
|---|---|---|---|
| 1 | $1.74 \cdot 10^{-8}$ | $1.21 \cdot 10^{-9}$ | $1.00 \cdot 10^{-7}$ |
| 2 | $3.87 \cdot 10^{-8}$ | $2.58 \cdot 10^{-9}$ | $1.00 \cdot 10^{-7}$ |
| 3 | $6.84 \cdot 10^{-8}$ | $4.91 \cdot 10^{-9}$ | $1.00 \cdot 10^{-7}$ |
| 4 | $3.75 \cdot 10^{-8}$ | $1.75 \cdot 10^{-9}$ | $1.00 \cdot 10^{-7}$ |
| 5 | $3.95 \cdot 10^{-8}$ | $2.50 \cdot 10^{-9}$ | $1.00 \cdot 10^{-7}$ |
| 6 | $5.32 \cdot 10^{-8}$ | $4.78 \cdot 10^{-9}$ | $1.00 \cdot 10^{-7}$ |
| 7 | $3.51 \cdot 10^{-8}$ | $2.53 \cdot 10^{-9}$ | $1.00 \cdot 10^{-7}$ |
| 8 | $3.88 \cdot 10^{-8}$ | $2.09 \cdot 10^{-9}$ | $1.00 \cdot 10^{-7}$ |
| 9 | $3.40 \cdot 10^{-8}$ | $1.75 \cdot 10^{-9}$ | $1.00 \cdot 10^{-7}$ |
| 10 | $3.77 \cdot 10^{-8}$ | $2.34 \cdot 10^{-9}$ | $1.00 \cdot 10^{-7}$ |
| 11 | $2.62 \cdot 10^{-8}$ | $1.50 \cdot 10^{-9}$ | $1.00 \cdot 10^{-7}$ |
| 12 | $5.55 \cdot 10^{-9}$ | $4.45 \cdot 10^{-10}$ | $1.00 \cdot 10^{-7}$ |
| 13 | $3.24 \cdot 10^{-8}$ | $2.07 \cdot 10^{-9}$ | $1.00 \cdot 10^{-7}$ |
| 14 | $2.88 \cdot 10^{-8}$ | $1.94 \cdot 10^{-9}$ | $1.00 \cdot 10^{-7}$ |
| 15 | $3.13 \cdot 10^{-8}$ | $1.84 \cdot 10^{-9}$ | $1.00 \cdot 10^{-7}$ |
| 16 | $1.04 \cdot 10^{-7}$ | $6.25 \cdot 10^{-9}$ | $1.00 \cdot 10^{-7}$ |
| 17 | $4.37 \cdot 10^{-8}$ | $2.55 \cdot 10^{-9}$ | $1.00 \cdot 10^{-7}$ |

**Table 5.** Parameters used in the multicomparment Darcy model: permeabilities. The parameters have been estimated in[18].

| Region | $\beta_{1,2}$ 1/(Pa s) | $\beta_{2,3}$ 1/(Pa s) | $\alpha$ m$^3$/(Pa s) |
|---|---|---|---|
| 1 | $4.93 \cdot 10^{-6}$ | $1.26 \cdot 10^{-6}$ | $4.65 \cdot 10^{-5}$ |
| 2 | $2.45 \cdot 10^{-5}$ | $2.83 \cdot 10^{-6}$ | $1.07 \cdot 10^{-4}$ |
| 3 | $3.06 \cdot 10^{-5}$ | $5.61 \cdot 10^{-6}$ | $3.15 \cdot 10^{-4}$ |
| 4 | $1.09 \cdot 10^{-5}$ | $2.02 \cdot 10^{-6}$ | $1.28 \cdot 10^{-4}$ |
| 5 | $1.91 \cdot 10^{-5}$ | $3.16 \cdot 10^{-6}$ | $1.63 \cdot 10^{-4}$ |
| 6 | $1.69 \cdot 10^{-5}$ | $3.16 \cdot 10^{-6}$ | $4.46 \cdot 10^{-4}$ |
| 7 | $1.08 \cdot 10^{-5}$ | $1.47 \cdot 10^{-6}$ | $2.22 \cdot 10^{-4}$ |
| 8 | $2.88 \cdot 10^{-5}$ | $4.14 \cdot 10^{-6}$ | $2.56 \cdot 10^{-4}$ |
| 9 | $5.07 \cdot 10^{-6}$ | $1.81 \cdot 10^{-6}$ | $4.91 \cdot 10^{-5}$ |
| 10 | $1.56 \cdot 10^{-5}$ | $2.06 \cdot 10^{-6}$ | $1.24 \cdot 10^{-4}$ |
| 11 | $5.28 \cdot 10^{-6}$ | $8.97 \cdot 10^{-7}$ | $7.23 \cdot 10^{-5}$ |
| 12 | $9.50 \cdot 10^{-7}$ | $1.97 \cdot 10^{-7}$ | $2.09 \cdot 10^{-5}$ |
| 13 | $7.69 \cdot 10^{-6}$ | $1.04 \cdot 10^{-6}$ | $8.22 \cdot 10^{-5}$ |
| 14 | $1.49 \cdot 10^{-5}$ | $2.04 \cdot 10^{-6}$ | $8.83 \cdot 10^{-5}$ |
| 15 | $9.90 \cdot 10^{-6}$ | $1.07 \cdot 10^{-6}$ | $5.51 \cdot 10^{-5}$ |
| 16 | $2.30 \cdot 10^{-4}$ | $1.47 \cdot 10^{-5}$ | $2.35 \cdot 10^{-4}$ |
| 17 | $3.41 \cdot 10^{-5}$ | $6.56 \cdot 10^{-6}$ | $1.12 \cdot 10^{-4}$ |

**Table 6.** Parameters used in the multicomparment Darcy model: pressure coupling coefficients ($\beta$) and coefficients in the Robin coupling condition ($\alpha$). The parameters have been estimated in[18].
